# Cyanobacteria and Alphaproteobacteria May Facilitate Cooperative Interactions in Niche Communities

**DOI:** 10.3389/fmicb.2017.02099

**Published:** 2017-10-25

**Authors:** Marc W. Van Goethem, Thulani P. Makhalanyane, Don A. Cowan, Angel Valverde

**Affiliations:** Centre for Microbial Ecology and Genomics, Department of Genetics, University of Pretoria, Pretoria, South Africa

**Keywords:** cyanobacteria, Namib Desert, hypolith, 16S rRNA, network analysis, keystone species

## Abstract

Hypoliths, microbial assemblages found below translucent rocks, provide important ecosystem services in deserts. While several studies have assessed microbial diversity of hot desert hypoliths and whether these communities are metabolically active, the interactions among taxa remain unclear. Here, we assessed the structure, diversity, and co-occurrence patterns of hypolithic communities from the hyperarid Namib Desert by comparing total (DNA) and potentially active (RNA) communities. The potentially active and total hypolithic communities differed in their composition and diversity, with significantly higher levels of Cyanobacteria and Alphaproteobacteria in potentially active hypoliths. Several phyla known to be abundant in total hypolithic communities were metabolically inactive, indicating that some hypolithic taxa may be dormant or dead. The potentially active hypolith network was highly modular in structure with almost exclusively positive co-occurrences (>95% of the total) between taxa. Members of the Cyanobacteria and Alphaproteobacteria were identified as potential keystone taxa, and exhibited numerous positive co-occurrences with other microbes, suggesting that these groups might have important roles in maintaining network topological structure despite their low abundance.

## Introduction

Extreme environmental conditions limit the occurrence of macroflora in deserts, resulting in more prominent roles for microbial communities in driving key biogeochemical processes (Makhalanyane et al., 2016). Specialized microbes are common in desert niches, such as those colonizing the ventral surfaces of rocks (Pointing and Belnap, 2012; Makhalanyane et al., 2015). In the zone below translucent rocks, referred to as the hypolithon, photoautotrophic, bacteria-dominated communities have crucial roles in biogeochemical processes by driving primary productivity (Friedmann and Ocampo, 1976) and biomass production (Chan et al., 2012). Photoautotrophs, such as Cyanobacteria, also contribute to soil carbon sequestration in deserts (Cockell and Stokes, 2004) while heterocystous species directly fix atmospheric nitrogen (Hutchins and Miller, 2017). There is direct evidence that these producers also mediate the transfer of nutrients and energy to heterotrophic bacteria (Tracy et al., 2010; Cowan et al., 2011). However, little is known regarding microbial community interactions, although it is recognized that these have implications for understanding biogeochemical cycling in soils (Valverde et al., 2015).

Hypoliths have been the focus of numerous studies on community assembly and microbial functionality owing to their relative trophic simplicity and pertinence in ecosystem service maintenance (Warren-Rhodes et al., 2006; Tracy et al., 2010; Azua-Bustos et al., 2011; Cowan et al., 2011). These studies have shown that hypolithic communities differ in composition from exposed desert soils (Makhalanyane et al., 2013; Stomeo et al., 2013) and other lithic communities, such as endoliths (Pointing et al., 2009; Van Goethem et al., 2016). Taxonomic differences across niches are mirrored at the functional level with recent studies highlighting the diverse nutrient cycling attributes in hypolithic communities in contrast to bare soils. These include, for instance, a wide spectrum of taxa driving nitrification and anammox pathways (Chan et al., 2013). Metagenomic studies have added to our understanding of hypolithic colonists by revealing numerous stress response mechanisms geared for coping with environmental challenges (Le et al., 2016; Vikram et al., 2016). Despite these advances, few studies have explored microbe–microbe interactions within hypolithic communities (Valverde et al., 2015). Such studies have the potential to reveal community dynamics and mechanisms of co-ordinating community function (Barberán et al., 2012; Shi et al., 2016). The spectrum of transcriptionally active taxa in hypolithic communities is also unknown.

Network analysis (measuring non-random co-variation patterns) is a powerful tool for exploring putative ecological associations among species (Dunne et al., 2002; Bissett et al., 2013). Studies of metacommunity networks have revealed the organization of microbial communities under different dispersal rates (Thompson and Gonzalez, 2017), and the inter-dependence of sub-communities that share metabolites, such as sugars and amino acids (Zelezniak et al., 2015). Microbial interactions within soil niche communities have also been explored recently (Barberán et al., 2012; Shi et al., 2016) and have shown that niche communities, such as the rhizosphere, are more complex in their interactions than surrounding soils. Defining the interactions that occur among active microorganisms is also important in understanding the factors that shape functional community dynamics (Gunnigle et al., 2017), particularly for identification of direct species interactions (Faust and Raes, 2012), or shared niches (Deng et al., 2012). Understanding community interactions has relevance for climate change predictions, for example, by indicating how the loss of low abundance microorganisms might alter community function (Lynch and Neufeld, 2015) and how the metabolic activity of important taxa might change (Tracy et al., 2010).

To better understand community interactions in niche environments, we used Illumina amplicon sequencing to assess the total and potentially active hypolithons. Total hypolithic community composition is represented by DNA-derived 16S rRNA gene sequences, whereas RNA-derived 16S sequences indicate protein synthesis potential at the time of sample collection (Blazewicz et al., 2013). We hypothesized that microbial taxa central to carbon and nitrogen fixation in desert niches, such as photoautotrophic Cyanobacteria (Chan et al., 2012), would be highly abundant in the active hypolithic community fractions.

Using this approach, we addressed the following questions: (i) Does the composition of potentially active hypolithic community members differ significantly from the total fraction of these communities? (ii) Are ecologically relevant microbes, such as photoautotrophic cyanobacteria, potentially active in hypoliths? and (iii) Which taxa serve as key components of potentially active hypolithic ecological networks?

## Materials and Methods

### Sample Collection

We collected cyanobacteria-dominated hypoliths on 15 April 2015 from an undisturbed site (23.558013 S, 15.039596 E) approximately 200 m from the Gobabeb Research and Training Station in the hyperarid central Namib Desert, Namibia. We sampled four hypolithic communities attached to the undersides of quartz rocks at three time points: in the morning (08h00), at midday (13h00), and at dusk (18h00) (*n* = 12, 3 time points × 4 replicates). All hypoliths sampled were similar in composition, size, and thickness. Samples were collected by scraping the adherent soil and microbial biomass into sterile 50 ml Falcon tubes using sterile razor blades. RNA was immediately stabilized by saturating the samples in LifeGuard^®^ Soil Preservation Solution (MoBio Laboratories, Carlsbad, CA, United States). Samples were stored at 4°C during transport to the laboratory, University of Pretoria (South Africa), and at -20°C until further processing.

### Nucleic Acid Extractions and Sequencing

Total microbial RNA was extracted from 2 g of hypolithic material from each sample (*n* = 12) using the RNA PowerSoil^®^ Total RNA Isolation Kit (MoBio Laboratories, Carlsbad, CA, United States) as per the manufacturer’s protocol. After extractions, RNA was eluted in 100 μl RNase/DNase-Free water and co-extracted DNA was digested at 37°C using DNase I (New England Biolabs, Ipswich, MA, United States). The absence of an amplification product following 16S rRNA gene PCRs using the e9F-u1510R primer set indicated that all genomic DNA had been digested (Baker et al., 2003). PCR amplifications were performed through an initial heating step at 95°C for 5 min, followed by 25 cycles of denaturation at 95°C (30 s), annealing at 57°C (30 s), and elongation at 72°C (90 s). We used the GoScript^TM^ Reverse Transcription System (Promega, Madison, WI, United States) for first-strand cDNA synthesis of microbial RNA. First-strand synthesis was performed following the manufacturer’s instructions, which required 2 μl of target RNA and amplification using the supplied random primers. All cDNA samples were stored at -80°C until required. Simultaneously, DNA was extracted from all hypolith samples (2 g) using a phenol-chloroform protocol optimized for soil samples (Miller et al., 1999). DNA was eluted in 50 μl autoclaved Millipore water and stored at -20°C until sequencing. Nucleic acids (cDNA and DNA) were sequenced on an Illumina MiSeq platform at the Host Microbiome Initiative (HMI), University of Michigan, United Staes.

### Sequence Processing

The 16S rRNA gene and transcript sequences were analyzed and quality processed in QIIME (v.1.9.1^1^) (Caporaso et al., 2010). Briefly, sequences were demultiplexed and poor-quality reads were removed if the phred score was below 20 for 6 consecutive bases. Chimeric sequences were detected and removed using USEARCH61 (Edgar, 2010). Prokaryotic OTUs (Operational Taxonomic Units) were clustered from sequences with 97% identity, representing the species level (Schloss and Handelsman, 2006), to UCLUST-based-open references and against the latest Greengenes database release 13_5 for 16S rRNA genes (DeSantis et al., 2006). All mitochondria and chloroplast sequences were removed from the analysis.

### Statistical Analyses

Statistical analyses were conducted in QIIME v.1.9.1 (Caporaso et al., 2010), R v.3.2.3 (R Foundation for Statistical Computing^2^), and STAMP (Statistical Analysis of Metagenomic Profiles^3^) (Parks and Beiko, 2010). All singletons were removed, and communities were randomly rarefied to 3,500 sequences per sample. The final high-quality datasets comprised 12 total (DNA) and 12 potentially active (cDNA) communities (samples). We used ANOVA to test for differences in α- (richness) and β-diversity (γ/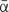) metrics.

Differences in microbial community structure were visualized using non-metric multidimensional scaling (nMDS) ordinations with Bray-Curtis distances of taxon relative abundances (Bray and Curtis, 1957). Using the ‘adonis’ function (PERMANOVA) in R (vegan package; Oksanen et al., 2013) we tested for significant differences in community structure between total and potentially active communities (DNA vs. cDNA) and by time of day (Morning vs. Midday vs. Dusk). Differences in within-group (DNA vs. cDNA) similarities was tested using ‘betadisper’ function, also in the vegan package. The number of unique and shared taxa were visualized using the venn function in R (gplots package). The proportion of potentially active and total taxa in each community were compared using STAMP, and the significant differences in taxon-relative abundances between these discreet groups were calculated using two-sided Welch’s *t*-tests. Statistical significance was assessed at α = 0.05 and where applicable, *P*-values were adjusted for multiple comparisons using Bonferroni correction.

### Co-occurrence Networks

Global network properties were calculated using the Molecular Ecological Network Analysis Pipeline (MENAP)^4^ (Deng et al., 2012). The connectivity of each node in the network was calculated using within-module connectivity (Zi) and among-module connectivity (Pi) scores which defined the topological role of each node (taxon). We classified our nodes as per the four categories describing node topology: network hubs (highly connected nodes within the entire network, Zi > 2.5 and Pi > 0.62), module hubs (highly connected nodes within modules, Zi > 2.5), connectors (nodes that connect modules, Pi > 0.62), and peripheral nodes (nodes connected in modules with few outside connections, Zi < 2.5 and Pi < 0.62) (Deng et al., 2012; Shi et al., 2016). We also created 100 random network graphs within MENAP using the same number of nodes and edges as the complete potentially active network to calculate random network characteristics. Cytoscape v.3.5.1 (Shannon et al., 2003) was used for visualization of significant co-occurrences, while the MCODE App v.1.4.1 was implemented to define modules (highly connected clusters) within the network (Bader and Hogue, 2003).

## Results

### Characteristics of Potentially Active and Total Hypolith Communities

**FIGURE 1:**
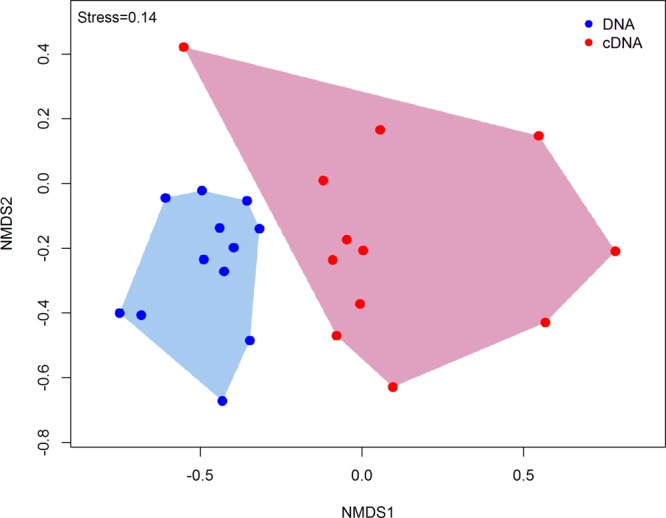
Non-metric multidimensional scaling (nMDS) ordination plot (Bray–Curtis dissimilarities) comparing the structures of total and potentially active hypolith communities. Each point within the ordination represents an entire microbial community (sample). Total hypolith communities are shown as blue circles while potentially active communities are indicated by red circles. Different shaded areas depict different bacterial communities (PERMANOVA, *P* < 0.05).

DNA and RNA amplicon sequencing showed significant differences in community composition between total (DNA) and potentially active (cDNA) hypoliths, respectively (**Figure 1**; PERMANOVA, *R*^2^ = 0.48, *P* < 0.001). The potentially active communities had significantly lower α-diversity and significantly higher β-diversity values compared to the total communities (ANOVA, *P* < 0.05; **Table 1**). Active and total communities also differed significantly in their dissimilarities, with active communities showing higher β-diversity (BETADISPER: *P* < 0.05).

**Table 1:** Diversity metrics for the potentially active (RNA) and total (DNA) hypolith communities.

Statistic	Total communities	Potentially active communities
Alpha-diversity ( 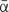 ), mean ± SD	523.6^a^ ± 89.8	195.33^b^ ± 90.2
Gamma-diversity (γ)	876	668
Beta-diversity (β) (γ/ 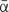 ), mean ± SD	1.72^a^± 0.11	4.15^b^± 0.58
Betadisper	0.33^a^	0.42^b^

*Different superscript letters indicate significant differences between potentially active and total communities (ANOVA, *P* < 0.05). Betadisper tests for differences in within-habitat dissimilarities (larger values indicate more dissimilar communities).*

**FIGURE 2:**
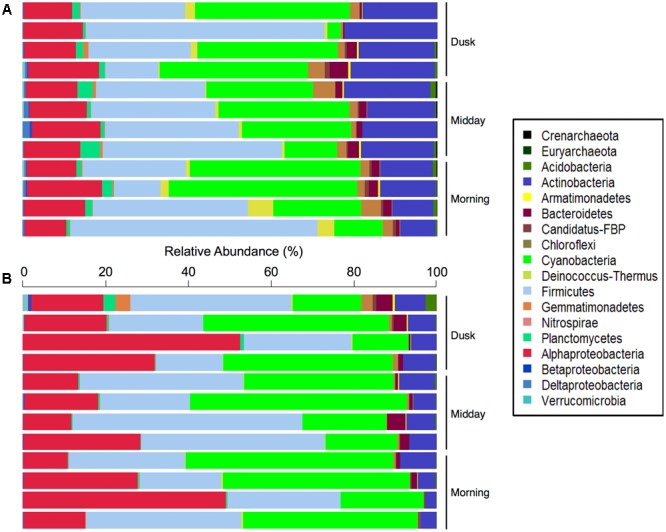
Relative abundances (%) of bacterial and archaeal phyla in the **(A)** total and **(B)** potentially active Namib hypolithic communities.

Overall, 14 bacterial and 2 archaeal phyla were identified, although only 7 of these phyla were at relative abundances above 1%. Firmicutes (32% of all DNA sequences) dominated the total hypolith communities, followed by members of Cyanobacteria (27%), Actinobacteria (16%), and Proteobacteria (14%). Minor bacterial phyla included Bacteroidetes, Chloroflexi, Deinococcus–Thermus, and Planctomycetes (**Figure 2**). A large percentage (22.8%) of the sequences could not be confidently classified at the genus level, suggesting that much of the microbial biodiversity in these desert soil niche communities has not yet been described (Anantharaman et al., 2016; Mukherjee et al., 2017). Overall, 128 microbial genera were found in the total hypolith fractions, 103 ± 8 genera per sample (mean ± SD), whereas 116 genera were found in the active fraction of the community (60 ± 14) (Supplementary Figure S1).

### Cyanobacterial Lineages Dominate Potentially Active Hypolithic Communities

The potentially active fractions of the communities were dominated by members of the Cyanobacteria (33% of cDNA sequences; **Figure 2B**), which produce carbon- and nitrogen-containing organic compounds such as amino acids, carbohydrates, and extracellular polymeric substances in hypoliths (Chan et al., 2012). This supports our hypothesis that species involved in carbon and nitrogen fixation in desert niches would be highly abundant in active hypolithic community fractions. The active Cyanobacteria spanned five orders, including Pseudanabaenales (25.4% of sequences assigned to Cyanobacteria), Synechococcales (22.5%), Oscillatoriales (14.6%), Chroococcales (14.4%), and Nostocales (1.4%), while 21.6% of cyanobacterial sequences could not be classified to a deeper taxonomic level. Other abundant bacterial phyla in the potentially active populations included heterotrophic Firmicutes (31%), Proteobacteria (25%), and Actinobacteria (6%), suggesting that members of these bacterial phyla may have important functional roles in these cryptic niches. A further 12 phyla were active in at least one sample, with Bacteroidetes (1.6%), Chloroflexi (0.59%), and Planctomycetes (0.52%) being the most prominent among these low abundance taxonomic groups.

**FIGURE 3:**
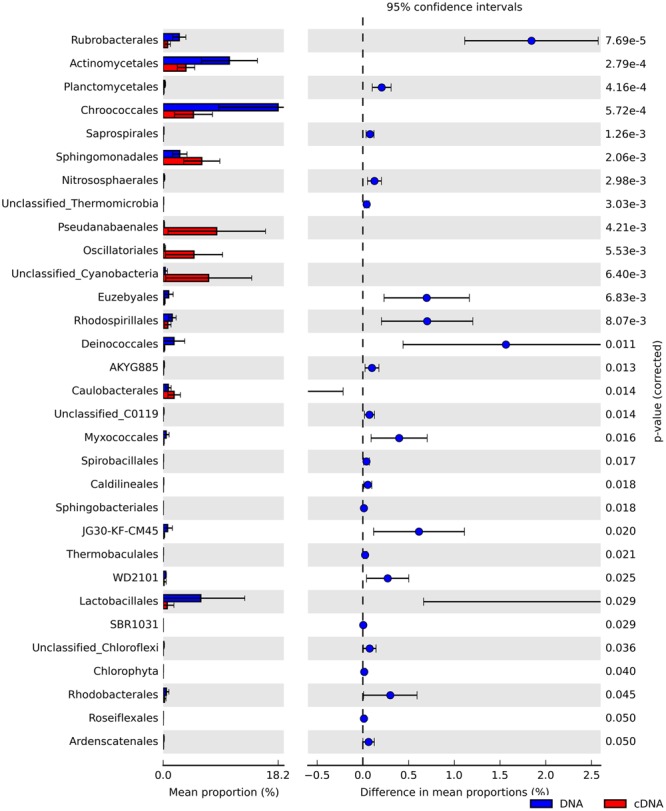
Extended bar charts showing significant differences in the relative abundances of taxa at the order level between the total (DNA, blue) and potentially active (RNA, red) hypolithic communities.

We identified numerous taxa that differed significantly in their relative abundance between the total and potentially active community fractions (**Figure 3** and Supplementary Figures S2, S3). At the phylum level, we noted that Actinobacteria, Chloroflexi, and Crenarchaeota were significantly enriched in the total community fractions compared to the active community fractions (two-sided Welch’s *t*-test; *P* < 0.05). We observed more significant differences (two-sided Welch’s *t*-test; *P* < 0.05) between the community types at finer taxonomic levels (Supplementary Figure S3). Members of the Oscillatoriales, Pseudanabaenales, and unclassified Cyanobacteria were significantly (two-sided Welch’s *t*-test; *P* < 0.05) over-represented in the RNA-derived communities (**Figure 3**). In contrast, members of the Chroococcales (Cyanobacteria) and Actinomycetales (Actinobacteria) were significantly under-represented in the potentially active communities relative to their total community counterparts (two-sided Welch’s *t*-test; *P* < 0.05). Since we did not sample at night, we cannot exclude the possibility that these taxa will become active under dark conditions.

Some compositional differences were related to time of sampling (Morning vs. Midday, Morning vs. Dusk) in the potentially active communities. Thus, members of the Planococcaceae (Firmicutes) significantly increased in their relative abundance, and potential activity, from morning to dusk (two-sided Welch’s *t*-test; *P* < 0.05). In direct contrast, members of the Paenibacillaceae (Firmicutes) produced more transcripts in the morning, and were significantly deceased in their transcript abundance at dusk (two-sided Welch’s *t*-test; *P* < 0.05). However, we found no significant differences in composition (PERMANOVA, *P* < 0.05 for all pairwise comparisons) between the total hypolithic microbial communities based on time of day (Morning vs. Midday vs. Dusk).

### Characteristics of the Potentially Active Network

Since habitat-filtering effects lessen the interpretability of co-occurrence networks (Berry and Widder, 2014), we opted to generate networks using only the active fractions of the communities. We used all time of day samples to generate networks, as few differences were related to time of day, and we observed highly similar network topologies from the three time points (data not shown).

**FIGURE 4:**
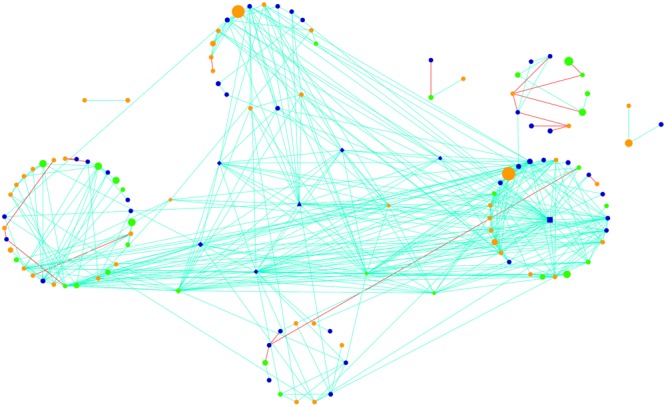
Global potentially active network comprising hypolithic community members at the genus level. Nodes represent Cyanobacteria (green), Proteobacteria (dark blue), and heterotrophic bacteria (orange). Lines connecting nodes (edges) are significant (*P* < 0.05) positive (light blue) or negative (red) relationships. Node size is proportional to the number of reads assigned to the taxon. Network hubs are triangles, module hubs are squares, connectors are shown as diamonds, and peripherals are indicated by circles.

The active community fraction network was highly modular in topology when evaluating networks generated using genus-level taxonomic assignments (**Figure 4**). After calculating various topological properties of the network (**Table 2**), including the type of co-occurrences (positive or negative) and connectivity, we identified five highly connected modules within the network (Supplementary Figures S4A–E and Table S1). Modules comprise highly connected taxa with fewer connections outside of the module, which potentially indicate a shared niche or hint at direct microbial interactions (Shi et al., 2016). Modules were mainly connected to each other by members of the Cyanobacteria and Alphaproteobacteria (Supplementary Figures S4A–E). Throughout the network we observed that taxa significantly co-occurred with each other (positive correlations, light blue lines) more often than they co-excluded (negative interactions, red lines; **Figure 4**). Strikingly, 95% of the potential interactions observed were positive correlations (**Table 2**) which could be interpreted as very high levels of cooperation between taxa driving core biogeochemical processes.

**Table 2:** Network statistics of the potentially active hypolith community network.

Topographical features	Active Network
Average node connectivity	13.01
Average path distance (GD)	3.58 (2.78)
Network diameter	9
Average clustering coefficient (avgCC)	0.338 (0.156)
Modularity	0.397 (0.293)
Connectedness (Con)	0.875 (0.983)
Transitivity (Trans)	0.409 (0.188)
Nodes	123
Edges (total)	414
Positive edges	394
Negative edges	20

*Values in brackets were obtained from 100 random networks (See “Materials and Methods”).*

### Putative Keystone Taxa

**FIGURE 5:**
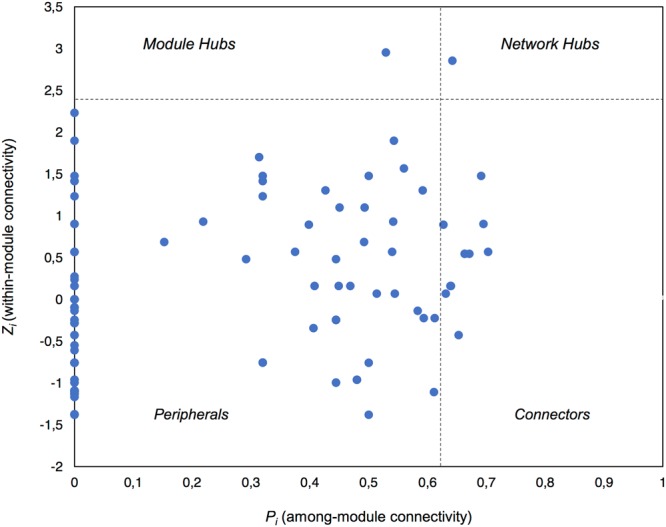
Plot showing the topological roles of the taxa in the complete active network. Each taxon is shown as a dot in the dataset. The topological role of each taxon was determined using the Zi (within-module connectivity) and Pi (among-module connectivity) scores in the scatterplot (Deng et al., 2012).

Using Zi (within-module connectivity) and Pi (among-module connectivity) scores (Deng et al., 2012), we found evidence of a network hub (an OTU belonging to Beijerinckiaceae), a module hub (an OTU belonging to Rhodobiaceae) and 10 connectors (**Figure 5**). Members of the Alphaproteobacteria class were the most common connectors (5 OTUs) as well as the network hub and module hub. Other connectors included members of the Cyanobacteria (3), Actinobacteria (2) and Bacteroidetes (1). Network hubs, module hubs, and connectors may represent keystone taxa within the network (Shi et al., 2016), interacting simultaneously with different active heterotrophic and phototrophic community members.

## Discussion

The results presented here should be considered with caution given that the methodology used has clear limitations. For instance, desiccation induces changes in the number of ribosomes, and the amount and quality of ribosomes varies with desiccation rates (Angeloni and Potts, 1986), which are expected to be different among the different components of bacterial communities, due, for example, to variation in the production of exopolysaccharides that contributes to water uptake and retention. The number of ribosomes can be high in dormant cells (Blazewicz et al., 2013 and references therein), which are expected to be numerous in deserts ecosystems (Lennon and Jones, 2011).

Notwithstanding the above, exploring the potentially active members of soil communities provides a more integrated understanding than DNA studies of the ecological roles played by microorganisms, by identifying which taxa may carry out biogeochemical cycling in the soil subsurface, and by documenting the putative interactions between potentially active members of the soil environment. Our results extend observations that potentially active and total soil microbial communities differ significantly in structure and composition (e.g., Urich et al., 2008). Comparable results were obtained using different soils from a variety of ecosystem types across the United States (Carini et al., 2016). We found that ca. 76% of hypolithic taxa potentially synthesize proteins at any given time point (Supplementary Figure S1), suggesting that a number of taxa in desert microbial communities are inactive, dead or dormant, and are not expected to contribute to community functionality. Dormancy is a life strategy common to all microbes, allowing non-replicative cells to remain viable during unfavorable conditions (Lennon and Jones, 2011; Lebre et al., 2017). Desert microorganisms are known to adopt dormancy strategies to avoid disturbance or resource limitation (Jones and Lennon, 2010; Dalling et al., 2011), which highlights the importance of identifying transcriptionally active species in desert niches.

Community membership differed significantly at many taxonomic levels between potentially active and total hypolith communities. We provide evidence that cyanobacteria of the orders Pseudanabaenales and Oscillatoriales are among the most active hypolithic community members. The over-representation of these cyanobacterial groups in the potentially active community fraction reflects their probable importance in the maintenance of desert soil carbon and nitrogen budgets (Warren-Rhodes et al., 2007). Heterotrophic bacteria in micro-niches are thought to benefit from the production and accumulation of organic substrates by cyanobacteria, and subsequently contribute to organic matter transformation and mineralisation (Chan et al., 2012).

However, not all cyanobacterial taxa present in the total community were prominent in the potentially active fraction, suggesting that only a subset of cyanobacteria may contribute to carbon and nitrogen fixation in these communities during a light cycle. The distribution, abundance and activity of cyanobacteria is strongly linked to environmental factors, such as temperature, water availability, resource availability, and light (Garcia-Pichel and Pringault, 2001; Garcia-Pichel et al., 2013; Rajeev et al., 2013), and therefore differences in how distinct species of cyanobacteria respond to these factors may explain these patterns. Indeed, we did not find members of the Chlorococcales to be among the most potentially active taxa, and in some Chlorococcales taxa (e.g., *Synechococcus* and *Prochlorococcus* sp.) the transcription of ribosomal genes appears to be higher during dark periods compared with light periods (Lepp and Schmidt, 1998; Zinser et al., 2009).

Network graphs, in which nodes represent taxa and edges reflect significant co-occurrences between taxa (Barberán et al., 2012), are a powerful tool to explore taxon coexistence within complex communities (Montoya et al., 2006; Eiler et al., 2012; Williams et al., 2014). The potentially active hypolithic network was highly modular in structure, in agreement with a previous study on ‘total’ Namib hypolith association networks (Valverde et al., 2015). Modular network structures have also been found in microbial rhizosphere assemblages (Shi et al., 2016) and in soil microbes subjected to long-term warming regimes (Deng et al., 2012). The modules found in the potentially active hypolithic network could potentially indicate direct or indirect interactions between microorganisms (Faust and Raes, 2012), but alternatively may be due to shared niches or a high level of phylogenetic relatedness (Lewinsohn et al., 2006). Modularity has been suggested to be an important attribute for ecosystem stability and community resilience, as disturbance events impact modular networks less severely than they do in non-modular structures (Olesen et al., 2007).

Based on ecological theory, each node in the network has a unique role in shaping the topology, which in turn influences the dynamics of the system (Guimera et al., 2007). Most of the taxa that were central to community structure belonged to the Alphaproteobacteria, mainly representatives of the Sphingomonadales and Rhizobiales, which have been suggested to be sources of phototrophy in deserts (Pointing et al., 2009) and nitrogen fixation (Black et al., 2012), respectively. This is particularly noteworthy as most of these nodes had low abundances in the active communities, which may indicate disproportionately large roles for these taxa in the hypolithic niche (Berry and Widder, 2014). These low abundance taxa had multiple, strong, positive interdependencies with heterotrophic and phototrophic community members within the hypolithic niche (**Figure 4** and Supplementary Figures S4A–E). Here, positive microbe–microbe interactions may lead to the transfer of energy between trophic levels, or facilitate the cycling of soil nutrients by multiple taxonomic groups (Steele et al., 2011; Berry and Widder, 2014). A similar result has been found in peatland communities, whereby an extremely rare sulfate-reducing bacterium was the most important functional member (Pester et al., 2010).

Positive co-occurrences were the dominant network connections (**Figure 4**). Supporting evidence suggests that positive interactions are relatively more abundant in desert ecosystems than in temperate biomes as microbial competition (negative interactions) is reduced (Fierer et al., 2012). This could be interpreted as a result of functional interdependencies among taxa or shared productivity under similar environmental stress conditions (Eiler et al., 2012). Soil communities from the hyperarid Atacama Desert also showed an extreme bias toward positive interactions, which indicated that correlated microbes responded similarly to environmental conditions (Neilson et al., 2017).

The loss of highly connected taxa may lead to the collapse of networks into disconnected sub-networks (Dunne et al., 2002), which has implications for community functionality. For example, the disappearance of keystone species may disturb community structure, potentially by disrupting important community functions that are carried out jointly by multiple species (Delgado-Baquerizo et al., 2016). Berry and Widder (2014) noted that the loss of a keystone species from simulated microbial communities directly affected co-occurring species that had positive interactions with keystone taxa. The loss of single functional groups can have detrimental impacts on community functionality, as soil communities experiencing losses in denitrifier diversity were shown to have reduced denitrification activity (Philippot et al., 2013). It is likely that the loss of active keystone members from hypolithic communities would have negative impacts on both the nature of community interactions and the biogeochemical processes that are supported in these niches.

## Conclusion

We found that Namib Desert hypoliths seem to be transcriptionally active, but that around 25% of the taxa are in states of apparent quiescence. This further supports recent findings (Carini et al., 2016) stating that the analysis of microbial community DNA alone is a highly unreliable guide as to the composition of active microbial populations. Most transcripts were attributed to photosynthetic Cyanobacteria of the orders Oscillatoriales, Pseudanabaenales, and Synechococcales. Members of the Alphaproteobacteria were also prominent in the potentially active fraction of these communities, which suggests that both Cyanobacteria and Alphaproteobacteria may be central to biogeochemical cycling in these niches. Using ecological network analysis, we identified several keystone taxa within potentially active hypoliths, which are most probably important in maintaining hypolithic community structure. Co-occurrence network analysis emphasizes the predominantly positive relationships among potentially active microorganisms, which we suggest is an important component of community structure and organization.

## Data Accessibility

The sequence data used in this study are available via the NCBI Sequence Read Archive under the BioProject number 395861.

## Author Contributions

MWVG conducted the analyses and wrote the first draft of the manuscript. DAC contributed formulation of ideas and edited the manuscript. AV and TPM conceived the project, assisted with analyses and edited the manuscript.

## Conflict of Interest Statement

The authors declare that the research was conducted in the absence of any commercial or financial relationships that could be construed as a potential conflict of interest.

## Footnotes

1 http://qiime.org/

2 https://www.r-project.org

3 http://kiwi.cs.dal.ca/Software/STAMP

4 http://ieg2.ou.edu/MENA/
