# Supplementary material for: Cyanobacteria and Alphaproteobacteria May Facilitate Cooperative Interactions in Niche Communities
Source: Front Microbiol. 2017 Oct 25;8:2099. doi: 10.3389/fmicb.2017.02099 (PMC5660985; doi:10.3389/fmicb.2017.02099)
Supplement: Supplementary file 1 [file Data_Sheet_1.pdf]

Cyanobacteria and Alphaproteobacteria may facilitate cooperative interactions in niche communities

Marc W. Van Goethem, Thulani P. Makhalanyane*, Don A. Cowan & Angel Valverde†*

Centre for Microbial Ecology and Genomics (CMEG), Department of Genetics, University of Pretoria, Pretoria 0028, South Africa

†Current address: Department of Microbial, Biochemical and Food Biotechnology, University of the Free State, Bloemfontein 9300, South Africa

*Correspondence: Thulani.makhalanyane@up.ac.za; avalverdeportal@gmail.com

Key words: Cyanobacteria, Namib Desert, hypolith, 16S rRNA, network analysis, keystone species

List of Supplementary Figures and Tables

Figure S1. Venn diagram indicating the distribution of taxa between total and potentially-active hypolithic communities.

Figure S2. Significant differences (two-sided Welch's t-test; P < 0.05) in the relative abundances of microbial taxa between total (DNA, blue) and potentially-active (RNA, red) communities at the (A) domain and (B) phylum levels.

Figure S3. Significant differences (two-sided Welch's t-test; P < 0.05) in the relative abundances of microbial taxa between total (DNA, blue) and potentially-active (RNA, red) communities at the family level.

Figure S4. Highly-connected clusters (A-E) identified using the MCODE application within Cytoscape. Nodes represent *Cyanobacteria* (green), *Proteobacteria* (dark blue), heterotrophic bacteria (orange). Lines connecting nodes (edges) are significant ($P < 0.05$) positive (light blue) or negative (red) relationships. Node size is proportional to the number of reads assigned to the taxon. Module hubs are squares, connectors are shown as diamonds, and peripherals are indicated by circles.

Table S1. Network statistics of the potentially-active hypolith sub-cluster networks.

Figure S1.

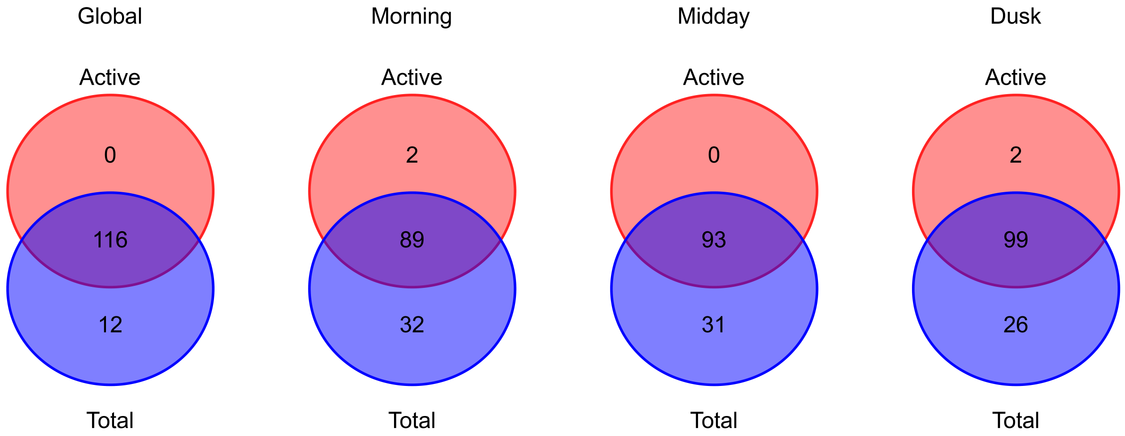
Global
Active
0
116
12
Total
Morning
Active
2
89
32
Total
Midday
Active
0
93
31
Total
Dusk
Active
2
99
26
Total

Figure S2.

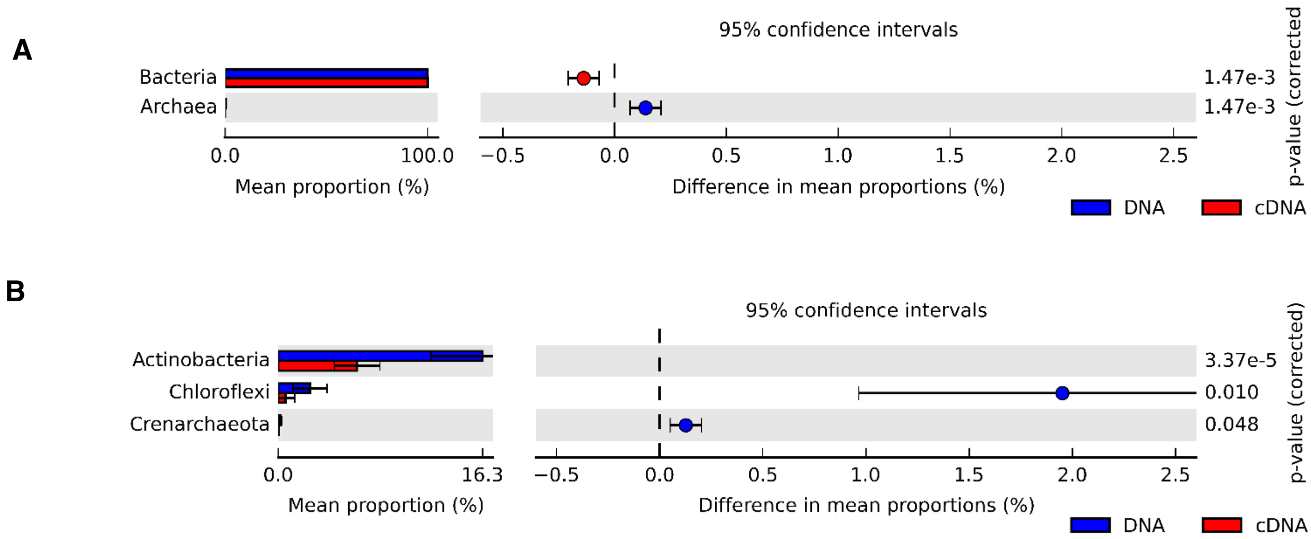
A
95% confidence intervals
Bacteria
Archaea
1.47e-3
1.47e-3
p-value (corrected
0.0
100.0
Mean proportion (%)
−0.5
0.0
0.5
1.0
1.5
2.0
2.5
Difference in mean proportions (%)
DNA
cDNA
B
95% confidence intervals
Actinobacteria
Chloroflexi
Crenarchaeota
3.37e-5
0.010
0.048
p-value (corrected)
0.0
16.3
Mean proportion (%)
−0.5
0.0
0.5
1.0
1.5
2.0
2.5
Difference in mean proportions (%)
DNA
cDNA

Figure S3.

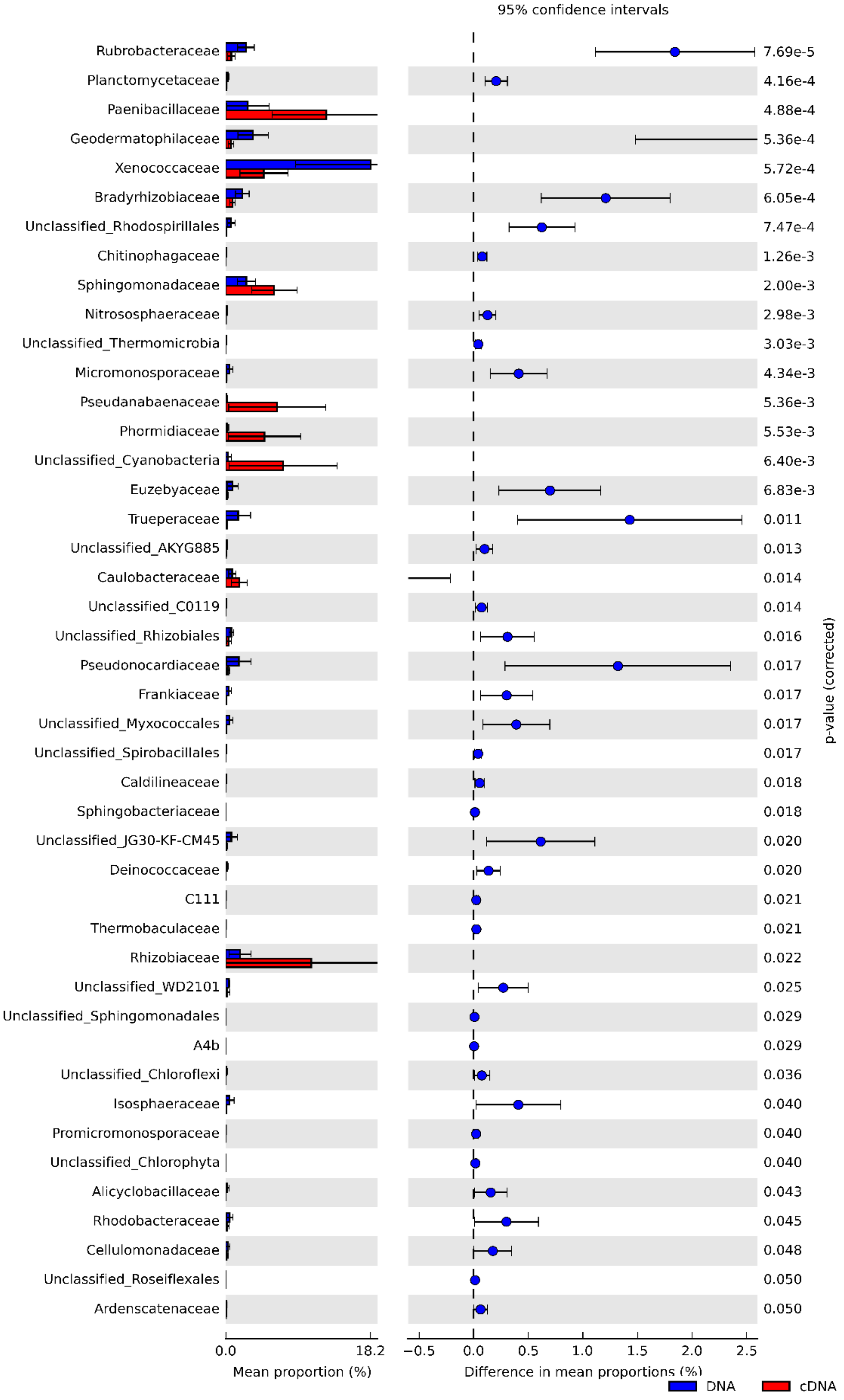
95% confidence intervals
Rubrobacteraceae
Planctomycetaceae
Paenibacillaceae
Geodermatophilaceae
Xenococcaceae
Bradyrhizobiaceae
Unclassified_Rhodospirillales
Chitinophagaceae
Sphingomonadaceae
Nitrososphaeraceae
Unclassified_Thermomicrobia
Micromonosporaceae
Pseudanabaenaceae
Phormidiaceae
Unclassified_Cyanobacteria
Euzebyaceae
Trueperaceae
Unclassified_AKYG885
Caulobacteraceae
Unclassified_C0119
Unclassified_Rhizobiales
Pseudonocardiaceae
Frankiaceae
Unclassified_Myxococcales
Unclassified_Spirobacillales
Caldilineaceae
Sphingobacteriaceae
Unclassified_JG30-KF-CM45
Deinococcaceae
C111
Thermobaculaceae
Rhizobiaceae
Unclassified_WD2101
Unclassified_Sphingomonadales
A4b
Unclassified_Chloroflexi
Isosphaeraceae
Promicromonosporaceae
Unclassified_Chlorophyta
Alicyclobacillaceae
Rhodobacteraceae
Cellulomonadaceae
Unclassified_Roseiflexales
Ardenscatenaceae
7.69e-5
4.16e-4
4.88e-4
5.36e-4
5.72e-4
6.05e-4
7.47e-4
1.26e-3
2.00e-3
2.98e-3
3.03e-3
4.34e-3
5.36e-3
5.53e-3
6.40e-3
6.83e-3
0.011
0.013
0.014
0.014
0.016
0.017
0.017
0.017
0.017
0.018
0.018
0.020
0.020
0.021
0.021
0.022
0.025
0.029
0.029
0.036
0.040
0.040
0.040
0.043
0.045
0.048
0.050
0.050
p-value (corrected)
0.0
18.2
Mean proportion (%)
−0.5
0.0
0.5
1.0
1.5
2.0
2.5
Difference in mean proportions (%)
DNA
cDNA

Figure S4.

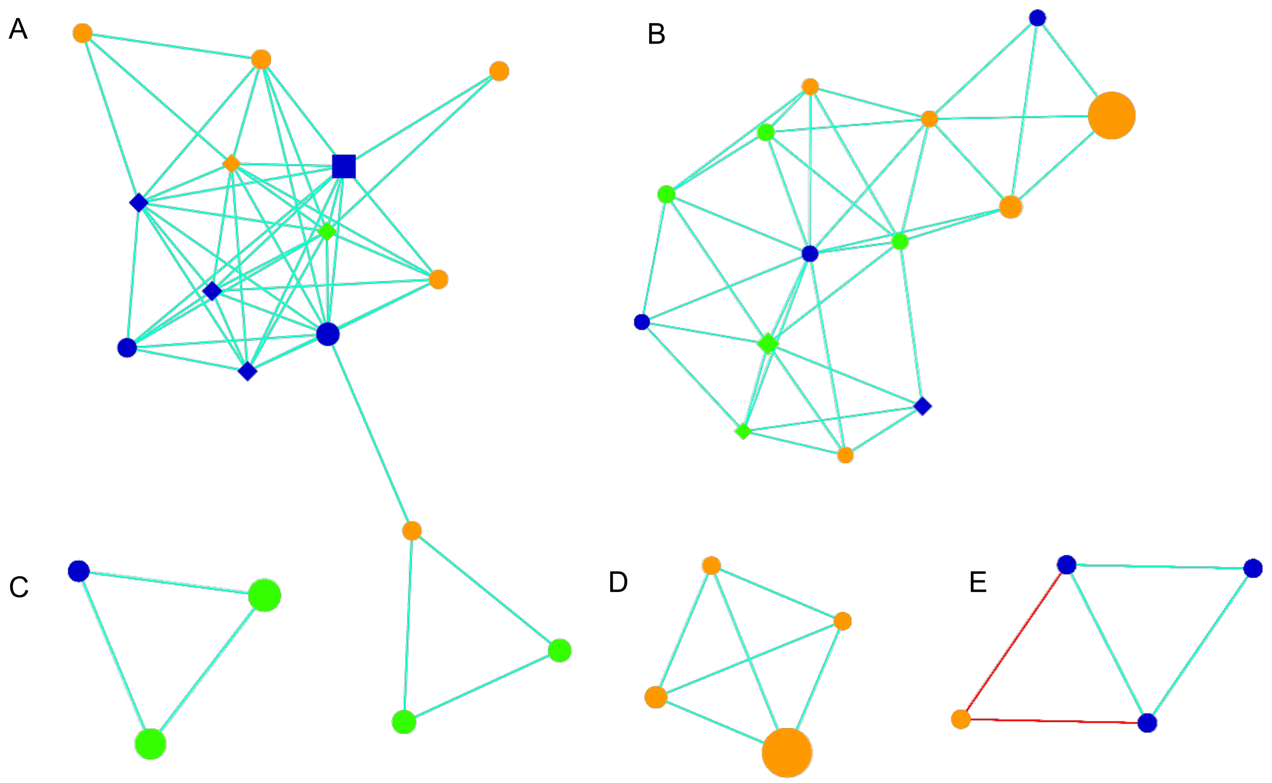

Table S1. Network statistics of the potentially-active hypolith sub-cluster networks.

Topological Features	Cluster A	Cluster B	Cluster C	Cluster D	Cluster E
Average node connectivity	7.33	5.75	2.67	-	-
Average path distance (GD)	1.83	1.73	1.17	1	1
Network diameter	4	3	2	1	1
Average clustering coefficient (avgCC)	0.786	0.697	0.833	1	1
Nodes	17	14	4	3	3
Edges (total)	60	37	5	3	3
Positive edges	60	37	3	3	3
Negative edges	0	0	2	0	0
